# Older Age and Larger Prostate Volume Are Associated with Stress Urinary Incontinence after Plasmakinetic Enucleation of the Prostate

**DOI:** 10.1155/2017/6923290

**Published:** 2017-03-30

**Authors:** Ning Xu, Shao-Hao Chen, Xue-Yi Xue, Yong Wei, Qing-Shui Zheng, Xiao-Dong Li, Jin-bei Huang, Hai Cai, Xiong-Lin Sun, Yun-Zhi Lin

**Affiliations:** Department of Urology, The First Affiliated Hospital of Fujian Medical University, Fuzhou 350005, China

## Abstract

*Background.* To investigate the factors associated with the occurrence of and recovery from stress urinary incontinence (SUI) after plasmakinetic enucleation of the prostate (PKEP).* Materials and Methods.* This retrospective study enrolled 1,288 patients with benign prostatic hyperplasia treated with plasmakinetic enucleation from January 2008 to January 2015, collecting demographics and clinical parameters. SUI was defined as a patient complaint of involuntary urine leak, including stress or mixed urinary incontinence. Logistic regression analysis was used to investigate the factors associated with the occurrence of SUI.* Results.* SUI after PKEP occurred in 80 of 1,288 patients (6.2%), 73 of whom (91.3%) recovered within 3 months and 78 of whom (97.5%) recovered within 6 months. In multivariate regression analysis of factors that were significant in univariate analysis, the factors that were significantly associated with postoperative SUI were age ≥ 70 years (odds ratio [OR] = 9.239; 95% confidence interval [CI] = 4.616–18.495; *P* < 0.001) and prostate volume on transrectal ultrasound ≥ 90 mL (OR = 15.390; 95% CI = 8.077–29.326; *P* < 0.001).* Conclusions.* SUI occurred in 6.2% patients after PKEP and was associated with older age and larger prostate volume. We suggest that age and prostate volume be considered in preoperative candidate selection before PKEP to reduce the occurrence of postoperative SUI.

## 1. Introduction

Benign prostatic hyperplasia (BPH) is the most common condition affecting men after age 50, resulting in bothersome lower urinary tract symptoms attributed to bladder outlet obstruction [1]. Transurethral resection of the prostate (TURP) remains the established gold standard surgical therapy for BPH [2, 3]. However, TURP can cause severe complications, including frequent need for reoperation, blood loss, and transurethral resection syndrome [4]. To reduce these complications, various minimally invasive approaches have been developed, including TURP with bipolar energy, vaporization of the prostate with bipolar energy, Holmium laser enucleation of the prostate (HoLEP), and potassium titanyl phosphate (KTP) laser vaporization of the prostate [5].

By dissecting along the surgical capsule, endoscopic prostatic enucleation enables anatomic enucleation of entire prostate lobes in the same way that a surgeon's finger does during open prostatectomy (OP). Recently, HoLEP has been reported to be an attractive, minimally invasive alternative to OP, with comparable functional results [6]. However, the steep learning curve and expensive laser equipment required for HoLEP have prevented widespread adoption of this procedure in developing countries [7]. In China, the Gyrus Plasma Kinetic (PK) system, the first bipolar device, has become widely used in urology. Plasmakinetic enucleation of the prostate (PKEP) has been accepted as a safe and technically feasible procedure for managing prostatic adenomas of any size, causing few complications and requiring no additional equipment. The technique achieved results comparable with surgical treatment and causes fewer complications than does open prostatectomy, providing a low morbidity and a shorter hospital stay [8, 9].

Surgery for BPH accounts for 10.3% of all cases of SUI in men, even though many trials have reported excellent treatment outcomes after surgery for BPH [10]. Early, temporary stress urinary incontinence lasting for a few weeks to a few months is an important issue facing patients and urologists. The incidence of SUI has been reported to be 3.7–16.7% following OP [11–13] and <3% after TURP [14]. Transient urinary incontinence occurs in 1.3–40.7% of patients undergoing HoLEP [15–17]. The incidence of SUI after PKEP has been reported to be 3.5%–18.9% [18], and it is usually temporary. To date, there is limited available data regarding the prevalence of SUI after PKEP, with few studies analyzing the risk factors for it, especially those that affect its occurrence after hospital discharge.

In this retrospective study, we used our database containing the information from many patients to define the incidence of SUI after PKEP, including during outpatient follow-up. Furthermore, we sought to determine the factors that may help to predict SUI development.

## 2. Methods

### 2.1. Patients

The study was approved by the Medical Ethics Committee of the First Affiliated Hospital of Fujian Medical University (approval number: 2014044). All patients provided verbal informed consent. We retrospectively reviewed the medical records of patients with BPH who were treated with PKEP from January 2008 to January 2015. A total of 1323 patients with BPH underwent PKEP by a single urologist (Dr. Wei). The inclusion criteria were failure of medical therapy, maximum flow rate < 10 ml/s, International Prostate Symptom Score > 19, or urinary retention requiring catheter placement [19]. Exclusion criteria were preoperative prostate cancer, incontinence due to urinary tract infection or mixed incontinence SUI before surgery, including those wearing pads for security reasons without evidence of SUI (mostly for postvoid dribbling), associated comorbidities preventing surgery, urethral strictures, and patients without complete medical records or who were lost to follow-up. Prostate biopsy was performed before surgery if serum prostate-specific antigen level and/or digital rectal examination raised suspicions of prostate cancer. In total, 1,288 patients were enrolled.

The following patient demographic and clinical information were collected: age, the history of diabetes or hypertension, preoperative medical treatment, body mass index, International Prostate Symptom Score, quality of life score, prostate volume on transrectal ultrasound, max flow rate, presence or absence of bladder stone, postvoid residual urine volume, operative time, enucleation time, resected weight, hospitalization time, and catheter indwelling time.

### 2.2. Surgical Procedures

All procedures were performed by a single urologist (Dr. Wei) in our institution. For patients receiving antiplatelet therapy, low-dose aspirin and/or clopidogrel was temporarily stopped five days before surgery and was resumed 3 days after surgery. Low molecular weight heparin was substituted in patients who had been taking warfarin. General or spinal anesthesia was administered in each case, and all patients were placed in the lithotomy position. All procedures were performed using a 27 Fr continuous flow resectoscopy (Karl Storz, Tuttlingen, Germany) and the PlasmaKinetic SuperPulse System (Gyrus Medical, Cardiff, United Kingdom), with a cutting power of 160 W and a coagulating power of 80 W. Physiologic saline was used for irrigation. The procedure was performed in a routine manner, and the detailed procedure has been previously described [9, 18, 20].

The bladder neck, verumontanum, and ureteral orifices were observed first, after which an incision was made close to the verumontanum in order to incise the urethral mucosa deep to the level of the surgical capsule. The tip of the resectoscope was inserted from the circular groove at the 5-o'clock and 7-o'clock positions to make a cleavage plane between the adenoma and the surgical capsule. The entire adenoma was then spun-off 360° from the surgical capsule, remaining attached only to the bladder neck in the 6-o'clock position. The denuded blood vessels and hemorrhagic spots on the capsule surface were identified and coagulated to block the blood supply to the lobe. The devascularized adenoma could then be rapidly resected in pieces using the cutting loop. When the resection was complete, all adenoma fragments were extracted using an Ellik evacuator, and a 20 Fr 3-way Foley catheter was placed and connected to straight drainage until hematuria resolved sufficiently. After December 2014, a morcellation device (Minitech Corporation, Guangzhou, China) was used to morcellate adenoma tissue after the enucleation was complete, pushing the enucleated tissue into the bladder in some patients.

### 2.3. Follow-Up

The presence of incontinence was recorded by the same urologist, based on the medical history obtained at the follow-up visit or by a telephone interview. Urinary incontinence was defined according to the recommendations of the International Continence Society [21]. In addition, to assess incontinence, patients were asked the following two questions: (1) “Does urine leak when you cough, sneeze, run, jump, lift weights, or go up the stairs?” and (2) “Does urine leak with a sudden, urgent desire to pass urine and loss of your bladder control before you can get to the bathroom?” The type of incontinence based on patient response was specified as being stress, urge, or mixed, for patients who responded positively to both the stress and urge questions. Follow-up evaluations, including assessment of incontinence, were performed for all patients at 2 weeks and 1, 3, 6, and 12 months postoperatively. In patients with SUI, recovery of continence was evaluated monthly after the problem was identified [22].

### 2.4. Statistical Analysis

Statistical analysis was performed using SPSS 19.0 statistical software (SPSS, Chicago, IL, USA). Quantitative data were compared using an independent samples *t*-test or the Mann–Whitney *U* test. Qualitative data were compared using an independent sample chi-square test or Fisher's exact test. SUI predictors were analyzed using logistic regression analysis and analyzed using a logistic regression model. A 2-tailed *P* value < 0.05 was considered statistically significant.

## 3. Results

The baseline characteristics and perioperative outcomes data of the whole cohort are shown in Table 1. SUI after PKEP occurred in 80 of 1,288 patients (6.2%), 73 of whom (91.3%) recovered within 3 months and 78 of whom (97.5%) recovered within 6 months. The remaining 2 patients (2.5%) had persistent SUI through 12 months of follow-up.

The patients were divided into 2 groups based on the absence (group 1, 1208 patients) or presence (groups 2, 80 patients) of postoperative SUI.  Table 2 compares the preoperative and intraoperative parameters of the groups. We found that age, prostate volume on transrectal ultrasound, quality of life score, total operative time, and total PSA were significantly associated with the development of SUI (Table 2). Multivariate regression analysis was used to evaluate factors that were significant in univariate analysis. Age ≥ 70 years (odds ratio [OR] = 9.239; 95% confidence interval [CI] = 4.616–18.495; *P* < 0.001) and prostate volume ≥ 90 mL (OR = 15.390; 95% CI = 8.077–29.326; *P* < 0.001) were significantly associated with postoperative SUI (Table 3).

## 4. Discussion

The goal of surgery for BPH is to improve quality of life by relieving micturition symptoms with a minimal risk of surgical complications [23]. Unfortunately, early SUI rates after PKEP vary from 3.5% to 18.9% [9, 18, 24]. This wide variation in the incidence of SUI may have arisen from the different definitions of postoperative SUI in each study [13]. We defined an involuntary urine leak as SUI, according to the recommendations of the International Continence Society [21], which includes stress or mixed urinary incontinence in the definition of SUI. However, some authors have only included a complaint of stress urinary incontinence in their definition. Although SUI after PKEP is known to be temporary in most cases, it remains among the most bothersome of postoperative complications following PKEP, for both patients and urologists. Many trials have reported excellent treatment outcomes after surgery for BPH. However, even temporary SUI may limit the activities of patients as they resume their daily lives.

The relationship between prostate volume on transrectal ultrasound and urinary incontinence after prostate enucleation remains controversial. Rao et al. [25] retrospectively reviewed surgical complications and outcomes based on prostate size in patients with BPH treated with PKEP, dividing patients into three groups (40 ml, 40–80 ml, and 80 ml) according to prostate size on preoperative transrectal ultrasonography measurement. They found that larger prostates required a significantly longer operation time in PKEP, but prostate size did not affect the incidence of perioperative and postoperative complications or micturition improvement. Elmansy et al. [17] performed a retrospective analysis of 949 consecutive patients treated over a 10-year period with HoLEP by a single surgeon. They found that HoLEP results in stress urinary incontinence at a rate comparable with that of other surgical techniques for the treatment of BPH. The presence of diabetes mellitus, large prostate volume, and a greater reduction in postoperative prostate-specific antigen remained statistically significant for the development of stress urinary incontinence. Our study also found that a large prostate volume, ≥ 90 mL, was significantly associated with the occurrence of SUI. We believe this is because a large prostate requires a longer operative time and more manipulation by the resectoscope across the external sphincter, resulting in transient sphincter dysfunction. The compression, stretching, and tearing by the resectoscope during the operation, together with a more complete removal of the prostatic adenoma, likely contribute to weakening or stretching of the external sphincter [13].

We also analyzed the relationship between age and the incidence of SUI, finding that age ≥ 70 years was significantly associated with SUI, which is consistent with other reports. Bruschini et al. [26] evaluated a total of 125 patients with urinary incontinence following surgical treatment for BPH. Urethral sphincter insufficiency is the main cause of incontinence following surgery for BPH patients older than 70 years, since sphincter function declines with increasing age [27]. The sphincteric tissue may be more fragile and sparse, compared with that of younger patients, which may lead to increased susceptibility to operative damage caused by forcing the tissue [13]. Older age and large prostate volume seemed to cause postoperative SUI more often, as well as delays in recovery from this complication. These patients should be warned about the higher risk of postoperative incontinence when consent is obtained. More meticulous and gentle enucleation can be done during surgery to reduce postoperative incontinence. Furthermore, some selected patients can be treated by conventional TURP.

There are several limitations to our study. Firstly, there were no objective measurements, such as a pad test or voiding diary. Moreover, there was no urodynamic study to exclude patients with detrusor or sphincter disorders. Secondly, given that this was a retrospective study, it may have been subject to selection and observation bias.

## 5. Conclusions

Stress urinary incontinence occurred in 6.2% of patients after PKEP. Age ≥ 70 years and prostate volume ≥ 90 mL were associated with postoperative SUI. We suggest that age and prostate volume be considered in preoperative candidate selection before PKEP to reduce the occurrence of postoperative SUI.

## Figures and Tables

**Table 1:** Baseline characteristics and perioperative outcomes data of the whole cohort.

Variables	Mean ± SD (range) or median (range) or number of patients (%)
Age (yr)	68.6 ± 7.3 (53–88)
BMI (kg/m^2^)	23.9 ± 3.2 (16.1–38.2)
Prostate volume (cm^3^)	70.1 ± 32.1 (29.1–240.0)
PSA level (ng/ml)	5.7 ± 6.9 (0.6–39.2)
Diabetes	200 (15.5%)
Hypertension	552 (42.9%)
No. preop treatment (%)	
Blockers	592 (46.0%)
5-Reductase inhibitor	659 (51.2%)
Both	453 (35.2%)
International Prostate Symptom Score	
Voiding symptoms	15 (5–20)
Storage symptoms	14 (1–15)
Total score	23 (12–35)
Quality of life score	5 (2–6)
Max flow rate (ml/s)	6.7 ± 3.2 (0–18)
Postvoid residual urine (mL)	126.8 ± 262.2 (0–2000)
Operation time (min)	77.7 ± 44.6 (20–240)
Enucleation time (min)	18.5 ± 7.5 (10–40)
Resected weight (g)	42.4.7 ± 16.1 (17.9–154.5)
Hospital stay (d)	4.9 ± 2.0 (3–10)
Catheter time (d)	2.1 ± 1.4 (1–7)

BMI: body mass index, PSA: prostate specific antigen, No. preop treatment: number with each specific preoperative treatment.

**Table 2:** Baseline characteristics and perioperative data of the two groups.

Variable	Patients without SUI (*n* = 1208) Mean ± SD (range) or median (range) or number patients (%)	Patients with SUI (*n* = 80) Mean ± SD (range) or median (range) or no patients (%)	*P* value
Baseline characteristic			
Age (yr)			<0.001
<70	727 (60.2%)	10 (12.5%)	
≥70	481 (39.8%)	70 (87.5%)	
BMI (kg/m^2^)			0.853
<24	632 (52.3%)	41 (51.3%)	
≥24	576 (47.7%)	39 (48.8%)	
Diabetes	184 (15.0%)	16 (20.0%)	0.254
Hypertension	520 (43.0%)	32 (40%)	0.594
No. preop treatment (%)			
Blockers	560 (43.0%)	32 (40.0%)	0.269
5-Reductase inhibitor	616 (50.9%)	43 (53.8%)	0.633
Both	424 (35.1%)	29(36.3%)	0.835
Bladder stone	158 (13.1%)	9 (11.3%)	0.637
Prostate volume (ml)			<0.001
<90	948 (78.5%)	14 (17.5%)	
≥90	260 (21.5%)	66 (82.5%)	
International Prostate Symptom Score			
Voiding symptoms	15 (5–20)	13 (7–20)	0.847
Storage symptoms	14 (1–15)	13 (2–15)	0.055
Total score	23 (12–35)	19 (16–35)	0.078
Quality of life score	5.2 ± 0.8 (2–6)	5.0 ± 1.2 (2–6)	0.037
Max flow rate (ml/s)	6.7 ± 3.2 (0–15)	6.7 ± 4.3 (0–18)	0.947
Postvoid residual urine (ml)	124.2 ± 261.3 (0–2000)	164.9 ± 276.0 (0–1000)	0.179
Serum total PSA (ng/ml)	5.6 ± 6.8 (0.6–39.2)	8.3 ± 7.4 (1.0–22.3)	0.002
Learning period			0.122
<50 cases	45	5	
≥50 cases	1,164	74	
Operation time (min)	76.7 ± 44.2 (20–230)	93.9 ± 47.7 (30–240)	0.001
Enucleation time (min)	18.6 ± 7.6 (11–60)	17.9 ± 6.3 (10–35)	0.437
Resected weight (g)	42.2 ± 16.0 (19.9–154.5)	45.5 ± 16.4 (17.9–84.2)	0.069
Hospital stay (days)	4.9 ± 2.1 (3–9)	4.6 ± 2.0 (3–10)	0.195
Catheter time (days)	2.1 ± 1.5 (1–7)	2.0 ± 1.4 (1–6)	0.468

BMI: body mass index, PSA: prostate specific antigen, No. preop treatment: number with each specific preoperative treatment.

**Table 3:** Multivariate predictors of postoperative transient incontinence.

Variable	Odds ratio (95% CI)	*P* value
Age (<70 versus ≥70) (yr)	9.239 (4.616–18.495)	<0.001
Prostate volume (<90 versus ≥90) (ml)	15.390 (8.077–29.326)	<0.001
Total PSA (ng/ml)	1.017 (0.985–1.049)	0.307
Quality of life score	0.830 (0.643–1.071)	0.152
Total operation time (min)	0.999 (0.993–1.004)	0.685

PSA: prostate specific antigen.
